# The perilous consequences of bowel preparation: a case study with literature review of Boerhaave syndrome

**DOI:** 10.3389/fmed.2024.1303305

**Published:** 2024-03-11

**Authors:** Ruo-yu Gao, Xiao-lan Wei, Jin-feng Wu, Zhi-wei Zhou, Xi-qiu Yu

**Affiliations:** Department of Gastroenterology, Shenzhen Luohu People’s Hospital, Shenzhen, China

**Keywords:** Boerhaave syndrome, colonoscopy, bowel preparation, treatment option, mannitol

## Abstract

Colonoscopy is widely acknowledged as a prevalent and efficacious approach for the diagnosis and treatment of gastrointestinal disorders. In order to guarantee an effective colonoscopy, it is imperative for patients to undergo an optimal bowel preparation regimen. This entails the consumption of a substantial volume of a non-absorbable solution to comprehensively purge the colon of any fecal residue. Nevertheless, it is noteworthy to acknowledge that the bowel preparation procedure may occasionally elicit adverse symptoms such as nausea and vomiting. In exceptional instances, the occurrence of excessive vomiting may lead to the rupture of the distal esophagus, a grave medical condition referred to as Boerhaave syndrome (BS). Timely identification and efficient intervention are imperative for the management of this infrequent yet potentially perilous ailment. This investigation presents a case study of a patient who developed BS subsequent to the ingestion of mannitol during bowel preparation. Furthermore, an exhaustive examination of extant case reports and pertinent literature on esophageal perforation linked to colonoscopy has been conducted. This analysis provides valuable insights into the prevention, reduction, and treatment of such serious complications.

## Introduction

Boerhaave syndrome (BS) is an infrequent yet perilous ailment precipitated by an abrupt surge in negative intrathoracic pressure and intraesophageal pressure (1), culminating in an unanticipated esophageal perforation. Appropriate intervention is imperative to avert the elevated mortality rates linked to this serious condition (2, 3). Esophageal perforation exhibits a substantial fatality rate, which escalates to 75–89% when therapeutic measures are postponed beyond 48 h (4). Prior investigations have indicated that individuals afflicted with eosinophilic esophagitis, medication-induced esophagitis, ileus, Barrett’s esophagus, childbirth, seizure, weightlifting or experiencing recurrent vomiting are more susceptible to the development of BS (5–7). Herein, we report the case of BS due to intestinal preparation.

## Case presentation

**Figure 1:**
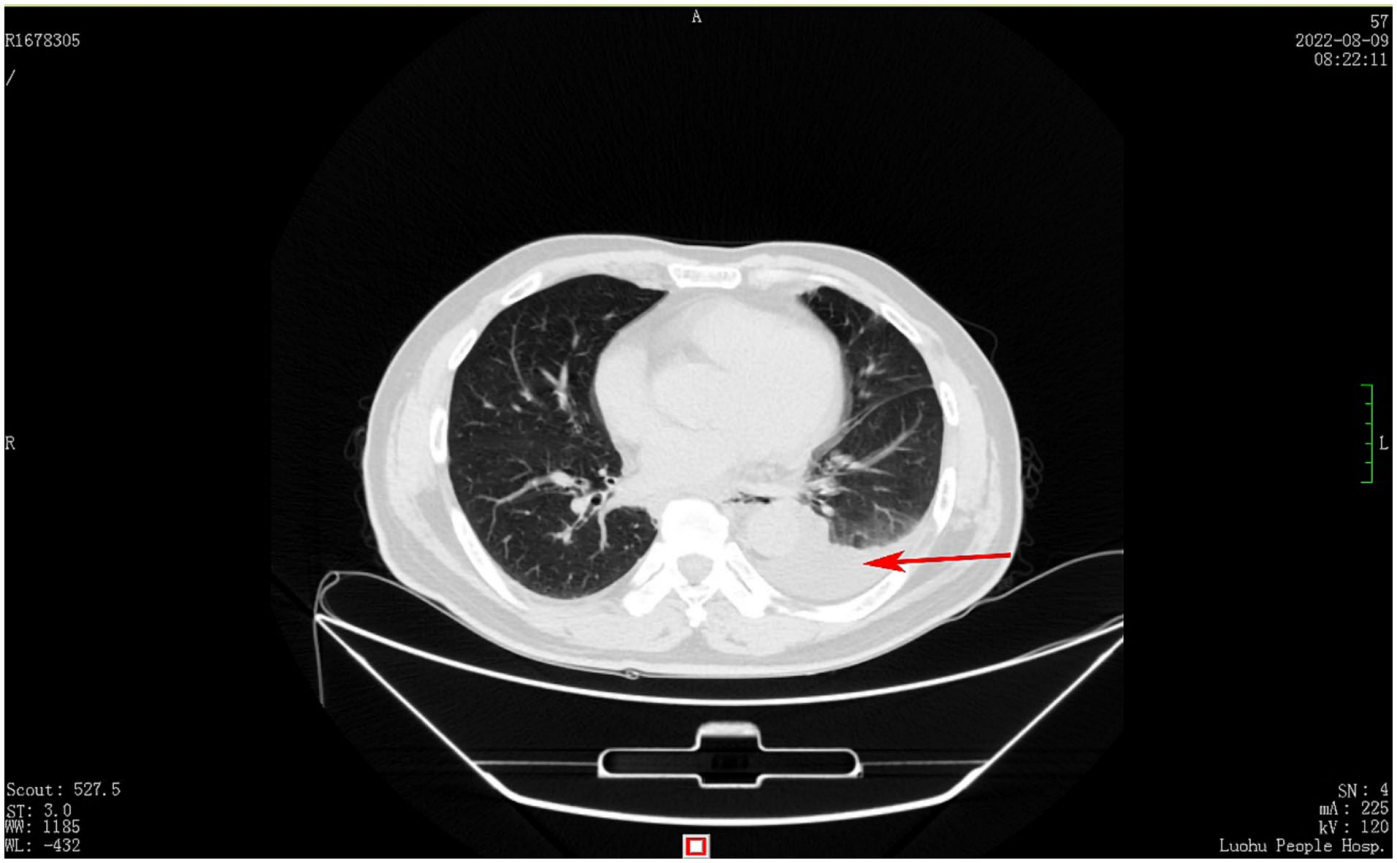
Chest computed tomography shows a left pleural effusion (red arrow).

**Figure 2:**
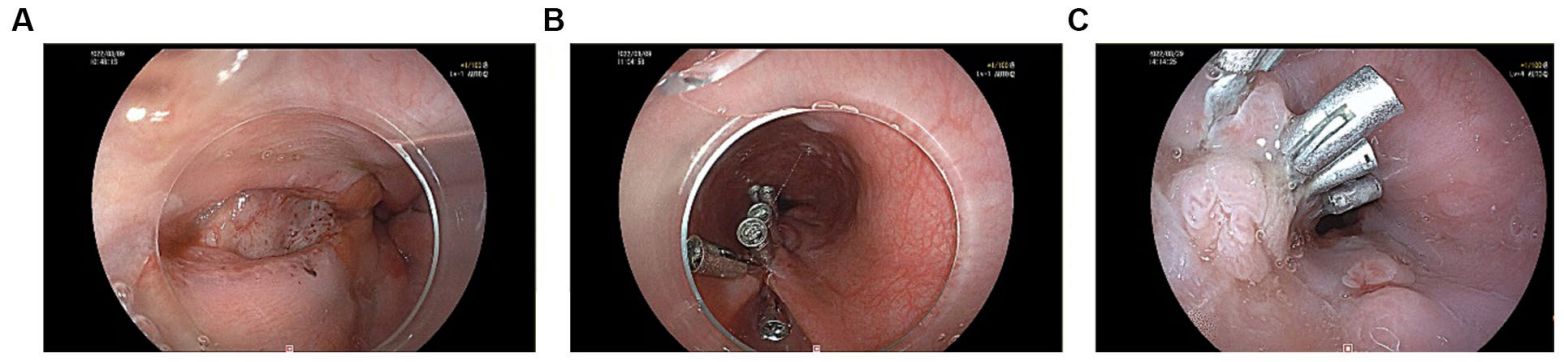
**(A)** A longitudinal laceration located in the esophagus was seen under gastroscopy. **(B)** Clamping the esophageal chasm with 6 titanium clips. **(C)** A well-closed, scar-like hyperplasia at the site of the primary rupture of the lower esophagus.

**Figure 3:**
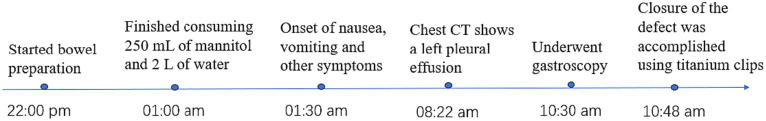
Time line of events.

**Figure 4:**
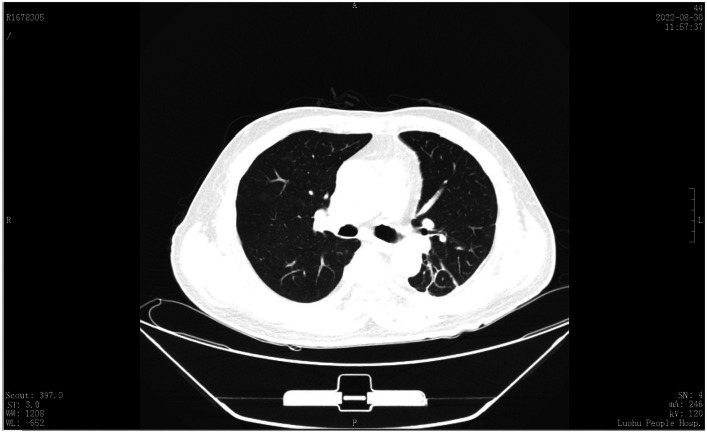
Recheck chest CT was showed pleural effusion disappeared 21 days after the operation.

A 60-year-old male patient, who was in a state of good health with no significant medical or familial history and no specific complaints, had been scheduled for a screening colonoscopy. The patient started bowel preparation at around 22:00 pm and finished consuming 250 mL of mannitol and 2 L of water at around 01:00 am. Half an hour later, the patient vomited twice and then developed symptoms such as upper abdominal pain, abdominal distension, and chest tightness. Upon physical examination, decreased breath sounds and dullness upon percussion were observed over the left lower lung without jugular venous distention and cardiac murmurs. Laboratory studies revealed elevated inflammatory markers [White Bloodcell Count (WBC) 16.88×109/L, hypersensitive C-Reactive Protein (hsCRP) 44 mg/L, Procalcitonin (PCT) 3.03 ng/mL]. Furthermore, biochemical examination showed no abnormal electrolytes or liver and renal function. A thoracic computed tomography (CT) scan revealed the presence of an esophageal-mediastinum fistula and pleural effusion on the left side (Figure 1). A diagnosis of BS was strongly suspected, leading to the immediate performance of gastroscopy in case of stable vital signs. The patient underwent gastroscopy at around 10:30 am, during the procedure, a longitudinal laceration (length approximately 50 mm) was observed in the esophagus, specifically located from 35 cm from the incisors to the cardia on the lesser curvature side, which was suspected to be the site of perforation (Figure 2A). Closure of the defect was accomplished using a total of 6 titanium clips (Figure 2B). Time line of events from start of prep to diagnosis and intervention as show as Figure 3. After surgery, patient was hemodynamically stable and transferred to the general ward. Following continuous left chest drainage, administration of anti-infection measures, and nutritional support, a re-examination of the gastroscope conducted 20 days after hospitalization revealed a well-closed, scar-like hyperplasia at the site of the initial rupture in the lower esophagus (Figure 2C). Repeat thoracic CT showed resolution of pleural effusion (Figure 4). Laboratory test revealed 5.06 × 10^9^/L WBC, 12 mg/L hsCRP and 0.04 ng/mL PCT, respectively. He recovered well and was discharged from hospital 3 weeks later.

## Discussion

We searched the literature in the databases Google Scholar, Medline, Web of Science, Embase, and Embase using keywords such as esophageal perforation, Boerhaave syndrome, bowel preparation, colonoscopy and complication. Few studies of esophageal rupture associated with colonoscopy and bowel preparation have been published (8–17). Herein we have reported these studies’ demographic features, symptoms, location of esophageal rupture, length of the laceration, diagnostic method, imaging findings, treatment and outcome in the following Table 1.

**Table 1:** Summary of reviewed cases.

References	Sex	Age	Underlying disease	Reasons for undergoing a colonoscopy	Bowel-cleansing agents	When	Symptoms	Location of esophageal rupture	Length of the laceration	Diagnosis	Primary treatment	Outcome	Hospital stay
Our case	Male	60	Not	Physical examination	250 mL of mannitol and 2 L of water	During the bowel preparation	Vomiting, abdominal pain, chest tightness	Left-sided distal	5 cm	CT: esophageal-mediastinum fistula and left-sided pleural effusion. Gastroscopy: laceration in the esophagus	Endoscopic titanium clip closure	Patient improved and discharged	3 weeks
Zhu et al. (8)	Male	48	Not	Physical examination	Unclear	During the colonoscopy procedure	Abdominal distension and shortness of breath	Left-sided distal	4 cm	CT: left-sided pleural effusion, mediastinal pneumatosis suspected perforation of the esophagus. Enhanced CT: contrast agent entering the chest from the esophagus, Meglumine diatrizoate esophagogram: esophageal perforation.	Thoracoscopic surgery	Patient improved and discharged	3 weeks
Xu et al. (9)	Male	63	Unclear	Unclear	Magnesium sulfate (Specific quantity unknown)	During the bowel preparation	Nausea, vomiting, epigastric pain, right chest compression pain, dyspnea, chest tightness and palpitation	Right-sided distal	Unclear	CT: mediastinal emphysema, right- sided pleural effusion and pneumothorax	Endoscopic titanium clip closure	Patient improved and discharged	Unclear
Nishikawa et al. (10)	Male	71	Unclear	Lower gastrointestinal bleeding 3 days after endoscopic mucosal resection for a cecal adenoma	2 L PEG	During the bowel preparation	Epigastralgia, dyspnea, and vomiting	Left-sided distal	Unclear	CT: mediastinal emphysema and left pleural effusion. Esophagography: Contrast media penetrated the mediastinal space and extended into the left thoracic cavity	Surgery	Patient improved and discharged	2 months
Yu et al. (11)	Male	61	Not	Hematochezia 24 h after resection of colonic polyp	2 L PEG	During the bowel preparation	Vomiting, chest pain and dyspnea	Left-sided distal	15 mm × 12 mm(could not be clearly demarcated due to blood)	CT: left pleural effusion and peri-esophageal fluid collection. Gastroscopy:15 mm × 12 mm perforation with stigmata of recent bleeding distal to the Z-line on the left side of the esophagus	Surgery	Patient improved and discharged	Unclear
Emmanouilidis et al. (12)	Female	73	Mild hypertension, a prosthetic hip joint, and colon diverticulosis	Physical examination	Prior to the day of examination: drinking the first 1,000 mL of MoviPrep solution. On the day of examination:400 mL PEG and appropriate additional amount of water	During the bowel preparation	Vomiting, back pain and abdominal pain	Left-sided distal	15 mm	CT with oral contrast medium: contrast medium extravasation at the level of the lower thoracic esophagus and mediastinal emphysema. Gastroscopy: a small longitudinal laceration (length approximately 15 mm) just above the Z line on the left side of the esophagus.	Surgery	Patient improved and discharged	3 weeks
Chalumeau et al. (13)	Male	72	Unclear	Physical examination	Unclear	During the colonoscopy procedure	Dyspnea, hypotension, tachycardia, and abdominal pain	Left-sided distal	Unclear	CT: left pneumothorax with pneumomediasti num. Radiography using water-soluble contrast: low esophageal perforation	Conservative therapy	Restarted eating by mouth 2 months after the colonoscopy	Unclear
Chalumeau et al. (13)	Female	75	History of untreated hiatal hernia	Physical examination	Unclear	During the colonoscopy procedure	Subcutaneous emphysema appeared	Distal	Unclear	CT: bilateral pneumothorax with pneumomediastinum. Radiographs using oral contrast: distal esophageal perforation	Surgery	Unclear	Unclear
Aljanabi et al. (14)	Male	85	Not	Per-rectal bleeding, alteration of bowel habits and soiling of 1 month duration	2 L PEG	During the bowel preparation	Vomiting, haematemesis, epigastric and lower chest pain, palpitation and dyspnoea	Unclear	Unclear	CT: bilateral moderately large pleural effusions, bilateral lower lobe atelactasis, fluid in the posterior mediastinum tracking up into the neck Omnipaque swallow: contrast extravasation into both pleural cavities from the distal esophageal region.	Conservative therapy	Died 36 h later	N/A
Eisen et al. (15)	Male	79	History of colon polyps	Hemalemesis	1 L PEG	During the bowel preparation	Chest pain, vomiting, hematemesis, chest and abdominal discomfort, shortness of breath	Left-sided distal	Unclear	Gastrografin study: esophageal leak	Surgery	Patient improved and discharged	11 days
McBride et al. (16)	Male	83	Appendectomy performed more than 40 years before	Positive stool occult blood	2 L PEG	During the bowel preparation	Chest pain and vomiting	Left-sided	1.5 cm	Esophagography: leak of the contrast medium from the distal esophagus into the left thoracic cavity	Surgery	Patient improved and discharged	3 weeks
Pham et al. (17)	Male	63	History of an asymptomatic hiatus hernia	Intermittent epigastric pain	2 L PEG	During the bowel preparation	Vomiting, chest and epigastrium pain	Left-sided	3 cm	X-ray after a barium swallow showed an esophageal leak.	Surgery	Patient improved and discharged	2 weeks

BS arises from a sudden increase in intraluminal pressure within the esophagus, resulting in a longitudinal laceration in the esophagus, with sizes ranging from 0.6 cm to 8.9 cm (18). During the process of bowel preparation, excessive intake of fluids can induce severe vomiting. At this point, pylorospasm may contribute to a delay in gastric emptying. Concurrently, powerful contraction of the abdominal and diaphragmatic muscles leads to a rapid elevation in intra-abdominal pressure. If esophageal spasms occur during the vomiting process, the gastric contents cannot be expelled, thereby causing a sudden rise in esophageal pressure and subsequent rupture. The anatomy of the esophagus is unique. In humans, the upper third is composed of skeletal muscle, the middle third is composed of mixed skeletal and smooth muscle, and only the distal third is composed of smooth muscle (19). Because of the presence of the smooth muscle layer at the distal esophagus, the tendency to retain food in this location and the fact that the distal esophagus lies below the left diaphragm, rupture of the esophagus is more likely to occur on the left side of the distal esophagus (20, 21).

Elderly persons are reported to have a higher risk of complications (such as cardiovascular/pulmonary complications, perforation, bleeding and mortality) during and after colonoscopy (22). However, it remains unclear whether esophageal perforation during the bowel preparation process is age-related. Previous reports and this case study have demonstrated a total of 9 cases experiencing esophageal rupture during bowel preparation and 3 cases during colonoscopy procedure. Based on our consolidated data, esophageal rupture during colonoscopy procedure and bowel preparation appears to be relatively more common in males (91.67%) and elderly persons (median age 71.5, range 48–85). The rupture is usually (in 90% of cases) in the lower third of the esophagus and in the left lateral position, longitudinal laceration sizes median 2.25 cm (ranging 1.5–5) (Table 1). One published case has been documented regarding esophageal rupture located in the right wall during the bowel preparation (9).

Once the esophagus is perforated, residual gastric contents, saliva, bile, and other secretions may enter the mediastinum, leading to chemical mediastinitis accompanied by mediastinal emphysema, inflammation, and subsequent mediastinal necrosis. Esophageal rupture is primarily manifested clinically as severe vomiting following binge eating or alcohol abuse, chest pain and tightness, radiating shoulder pain, dyspnea and respiratory distress, subcutaneous emphysema, fever, hematemesis and abdominal pain. Boerhaave syndrome is often misdiagnosed as peptic ulcer, acute pancreatitis, or myocardial infarction due to its rarity and nonspecific symptoms (1). Among the 12 integrated case reports in this article, 9 cases (75%) presented with vomiting, 7 cases (58%) experienced abdominal pain, 7 cases (58%) had difficulty breathing or shortness of breath, and 6 cases (50%) reported chest pain. We suggest that vomiting, abdominal pain, chest pain and difficulty breathing or shortness of breath be considered red flags, as they manifest in at least 50% of Boerhaave syndrome cases.

Chest X-rays, CT scans, esophagography, or endoscopic examinations can provide diagnostic information on esophageal perforation. On chest X-ray, findings suggestive of esophageal perforation include free gas in the peritoneal cavity, pneumomediastinum, or subcutaneous emphysema. However, chest X-ray has limited sensitivity for detecting esophageal perforation (5). For patients who are not suitable for invasive examinations, consider performing esophagography, as leakage of the contrast agent serves as a reliable indicator for confirming the diagnosis of perforation. The choice of contrast agents for esophagography include water-soluble contrast (such as meglumine diatrizoate) and barium contrast. Compared to water-soluble contrast, barium contrast has a better performance for smaller perforations, and it can detect 60–90% of esophageal perforations (23). However, due to the potential for barium contrast to cause mediastinal or pleural cavity inflammatory reactions, it is generally preferred to use water-soluble contrast (24). Additionally, chest CT scans are also an effective method for diagnosing esophageal perforation. A CT scan of the chest revealed periesophageal fluid accumulation, thickening of esophageal wall with oedema, effusion and gas in pleural and peritoneal cavities, compatible with an esophageal perforation (25–27). However, small esophageal perforations may present as negative findings on CT scans, as the swollen esophageal wall may close the fistula orifice. For stable patients with no suppurative complications, we can perform an upper gastrointestinal endoscopy (28). Endoscopy allows direct visualization of the specific site and size of the esophageal perforation. The role of endoscopy in the diagnosis of spontaneous esophageal perforation is controversial, as the endoscopic procedure can extend the perforation and introduce air into the mediastinum (29).

The mortality rate is high in cases of esophageal rupture. Early and definitive diagnosis leads to specific treatment, which can improve prognosis. In a report of 12 cases, 8 cases (66.7%) underwent surgical treatment, recovered well and was discharged from hospital 11 days to 2 months later. 2 cases (16.7%) underwent endoscopic titanium clip closure, our case showing improvement and being discharged after 3 weeks, and the other case showing significant improvement on chest CT a few days later, with no contrast agent leakage on esophagography (9). Two cases (16.7%) of patients with poor general condition were treated conservatively, and one of them died within 36 h after conservative management (14). The other patient restarted eating by mouth 2 months after the colonoscopy (13). For patients with esophageal rupture, it is crucial to develop urgent and personalized treatment plans. Treatment options include conservative management, endoscopic intervention, or surgical approaches. Conservative management is suitable for patients with mild symptoms or poor general condition who cannot tolerate surgery or endoscopic examination. For patients with stable conditions or those unlikely to tolerate surgery, endoscopic treatment should be considered (30–32). Common endoscopic interventions include self-expanding stents (included metal stents and plastic stents) (28, 33–35), endoscopic negative pressure therapy (ENPT) (36), endoluminal vacuum therapy (31), endoscopic suturing, esophageal resection, through-the-scope (TTS) clips, over-the-scope (OTS) clips and diversion (37). The treatment of patients should be personalized based on their condition, the extent of the disease, and the timing of symptom onset.

As a rare but serious complication, Boerhaave syndrome following colonoscopy procedure or bowel preparation may worsen the prognosis of patients. Therefore, several strategies have been proposed to reduce the esophageal rupture. PEG electrolyte solution is widely used for bowel preparation (38), and nausea is a common side effect (39). Due to the large volume of solution required for colon cleansing, which increases the likelihood of vomiting. Esophageal rupture is more commonly observed in elderly males, patients with esophagitis, ileus, Barrett’s esophagus, childbirth, seizure, weightlifting or esophageal hiatal hernia. Firstly, in this high-risk patient population, administering PEG electrolyte solution via a nasogastric tube prior to colonoscopy may help prevent this complication. We believe that anti-emetic medication during the bowel preparation process may potentially reduce the incidence of this complications in high-risk individuals (12). Additionally, patients should be advised to discontinue the consumption of PEG electrolyte solution if they experience vomiting. Secondly, we can reduce the complications by minimizing the intake of PEG. Relevant studies have indicated that there is no significant difference in bowel cleansing efficacy between orally consuming 1 L PEG with linaclotide and consuming 2 L PEG (40). However, patients in the 1 L PEG with linaclotide group reported less nausea and vomiting (40). In addition, slowing down the intake speed of liquids can also reduce the occurrence of such symptoms. Thirdly, we can choose bowel cleansing agents that are more better tolerated in elderly patients. There are various bowel cleansing agents available for bowel preparation, such as magnesium sulfate solution (MSS), magnesium citrate, lactulose, mannitol, sodium phosphate and sodium picosulfate (41–46). A study involving 1,174 patients demonstrated that low-dose of MSS is non-inferior to the standard PEG regimen in terms of bowel preparation quality for elderly and MSS offers fewer nausea, vomiting and better tolerability (41).

## Conclusion

For patients who experience nausea, vomiting, and subsequent chest or abdominal pain during the bowel preparation process or colonoscopy, we should promptly consider the possibility of spontaneous esophageal rupture. Early intervention and treatment can lead to favorable outcomes for patients. For certain high-risk individuals, appropriate measures can be taken to minimize the occurrence of such complications.

## Data availability statement

The raw data supporting the conclusions of this article will be made available by the authors, without undue reservation.

## Ethics statement

Written informed consent was obtained from the individual(s) for the publication of any potentially identifiable images or data included in this article.

## Author contributions

R-yG: Writing – original draft, Writing – review & editing. X-lW: Writing – review & editing, Writing – original draft. J-fW: Supervision, Visualization, Writing – review & editing. Z-wZ: Supervision, Writing – review & editing. X-qY: Supervision, Validation, Visualization, Writing – review & editing.
