# Supplementary material for: The perilous consequences of bowel preparation: a case study with literature review of Boerhaave syndrome
Source: Front Med (Lausanne). 2024 Mar 11;11:1303305. doi: 10.3389/fmed.2024.1303305 (PMC10961334; doi:10.3389/fmed.2024.1303305)
Supplement: Supplementary file 1 [file Presentation_1.PPTX]

## Slide 1
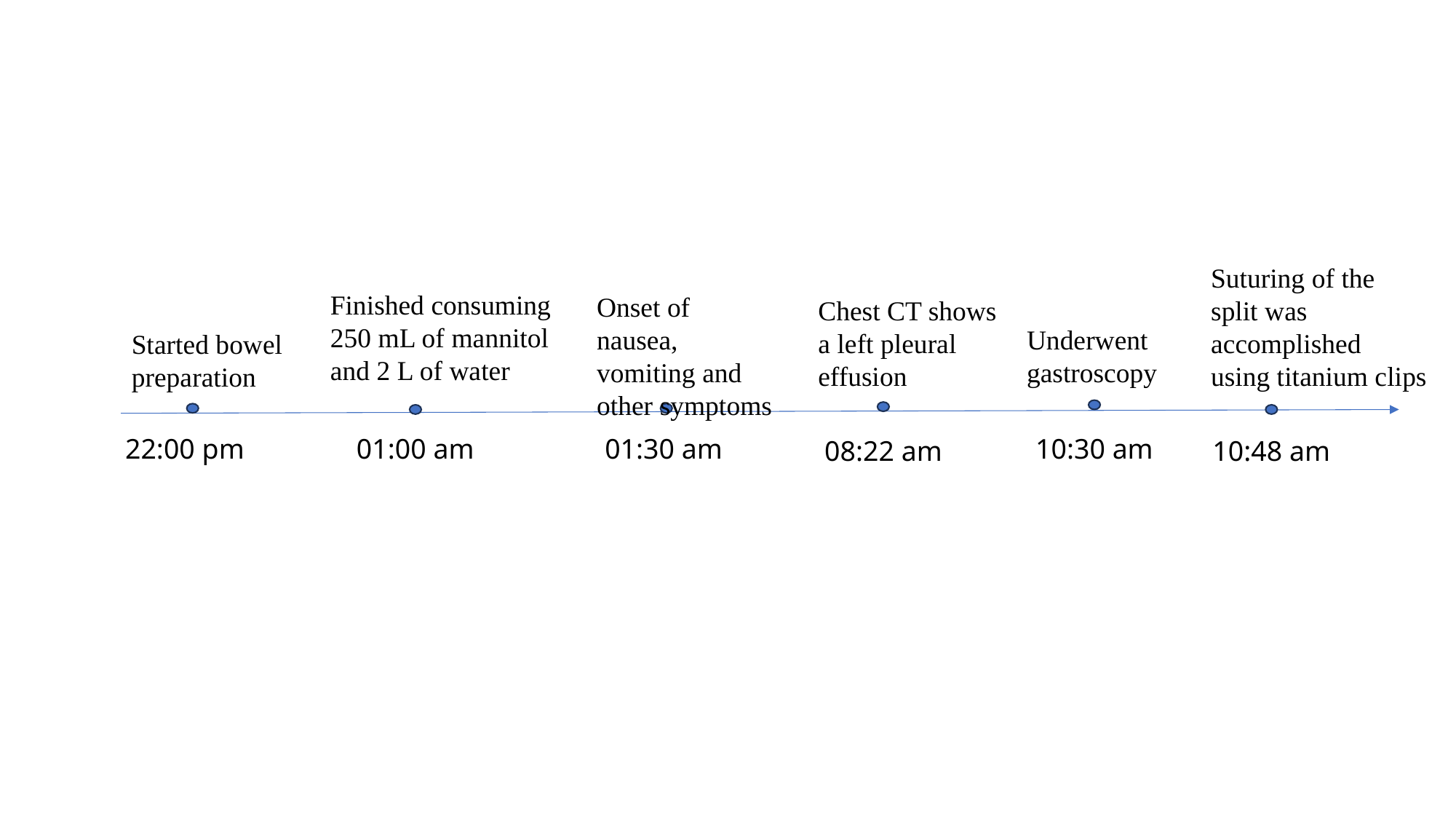

Suturing of the split was accomplished using titanium clips
Finished consuming 250 mL of mannitol and 2 L of water
Onset of nausea, vomiting and other symptoms
Chest CT shows a left pleural effusion
Underwent gastroscopy
Started bowel preparation
22:00 pm
01:00 am
01:30 am
10:30 am
10:48 am
08:22 am
